# Medicine, Value, and Knowledge in the Veterinary Clinic: Questions for and From Medical Anthropology and the Medical Humanities

**DOI:** 10.3389/fvets.2022.780482

**Published:** 2022-03-14

**Authors:** Jane Desmond

**Affiliations:** Department of Anthropology, College of Veterinary Medicine, University of Illinois at Urbana-Champaign, Urbana, IL, United States

**Keywords:** medical humanities, veterinary medicine, animal agency, consent, veterinary anthropology, multi-species ethnography, species valuation, differences between human and veterinary medicine

## Abstract

The welcome development of the veterinary humanities, and veterinary anthropology specifically, raises the question of its potential relationship with the now well-established field(s) of the medical humanities, and of medical anthropology. Although there are national variations, the term “medical humanities” generally refers to either the tapping of the humanities to improve medical education by developing, through engagement with the humanities like literature and visual art, skills in empathy, visualization and expressivity, or alternatively, it refers to the application of humanities approaches of cultural critique to the presumptions, practices and institutions of the human medical world to denaturalize the ideologies of knowledge that contemporary human medicine professions depend upon. This article reflects on the potential impact that the development of a veterinary medical humanities could have on the field of (human) medical humanities and vice versa. Could such a development force a re-conception of notions of agency, of consent, and of the position of “patient” when the (human based) medical humanities is expanded to include both human and veterinary medicine? What would the potential usefulness, or limitations, both in conceptual and in applied terms, be of constructing a multi-species notion of “medical humanities?” What can such a comparative approach offer to veterinary medicine, in practice and in terms of the curricula of veterinary training? To reflect on these questions, this article draws on my multiple years of fieldwork in veterinary clinics and classrooms to first lay out the constituent components of the formal practice of contemporary veterinary medicine (at least in the U.S.) in terms of the roles that species specificity and relations to humans play in the delivery of care, and then seeks to center the animal in these practices to ask questions about consent, resistance, veterinary obligation, and the role of finance in comparison with human medicine. These similarities and differences will form the basis for a consideration of the effects of enlarging the medical humanities to encompass more than one species.

## Introduction

Several years ago, when I was just starting my fieldwork in veterinary medicine, I found myself unexpectedly breathing for a duck. A resident beside me in the operating room suddenly thrust a blue breathing bag into my hands, with terse instructions before she dashed off: press the bag slowly, every 10 s. As I frantically counted, I watched in amazement as each pumping action of my palms inflated the feathered chest of the sedated Mallard—the direct connection between my body and his providing a literally life-sustaining infusion of oxygen. Dressed in scrubs as I was, the Resident hadn't known I wasn't a vet student watching the operation, but rather an anthropologist conducting fieldwork. Eventually she returned and reclaimed the breathing bag with a quick thanks. I'd done it! I hadn't missed a cycle, pressed too hard, too lightly, too slowly, too quickly. Even in a room full of doctors and doctors-to-be, I had felt the weight of that sudden responsibility for another life—a life so unlike mine. This avian being—with his iridescent feathers, hollow bones, ability to fly and to dive deep underwater—lived a life fundamentally different from mine—and yet here we were literally entangled by the narrow plastic tube connecting my hands and his throat.

In the interdisciplinary field of human-animal studies (1, 2), also called Animal Studies or anthrozoology, which unites work across the humanities, arts, and social sciences, scholars often speak of “entanglement” between human and non-human animals (3, 4). The term implies a deeply connected, almost inextricable set of conceptual, political and lived relationships, hard to trace in their complexity. In veterinary medicine this notion of entanglement is not only a metaphor that aims to capture complexity, but also a literal set of connections as well—from body to body-across the species line^1^. In this article, I ask how can we investigate, conceptualize and theorize these connections, especially in terms of clinical medicine? How they are similar to or different from human medicine. What available conceptual tools might be called on from other fields? What new ones might we need to develop?

To probe these questions, I start from an anthropological commitment to fieldwork as a method of deep engagement, over time, with a community. I draw on extensive fieldwork in veterinary clinics to chart the organization of veterinary medical practice in the U.S., the country to which I limit my remarks while acknowledging that animal doctoring is done around the world in different ways and by different types of specialists or lay practitioners. Then I consider the potential of the medical humanities to focus further investigations in veterinary medicine. And I suggest ways that new considerations can potentially enhance and enlarge current modes of veterinary training.

My fieldwork has been carried out primarily in the teaching hospital, labs, and classrooms of my home university, the University of Illinois at Urbana-Champaign, U.S.A. In addition, I have benefited from time spent at Colorado State University's College of Veterinary Medicine. The academic setting of my fieldwork has both strengths and limitations. On the one hand, it engages with the most current state-of-the-art training and with techniques described as “gold standard” medicine. These represent the mode of training that most U.S. veterinarians receive. On the other hand, it does not necessarily replicate the practice of medicine across a full spectrum of clinics outside of teaching hospitals.

A second framing choice has to do with species examples. While my fieldwork has involved rotations across the full range of clinical practice and training, from farm animal medicine, to companion animal medicine, to the diagnostic and anatomy labs, radiology, surgery suites, and emergency rooms, many of my examples will be drawn from interactions in clinics devoted to exotic and wildlife medicine. In this arena, many of the challenges of medicine are the same as other specialties: how to devise a “differential diagnosis, ” how to interact with owners/clients, choosing which tests to run when and why, and so on. On the other hand, this subspecialty strikingly reveals some of the most fundamental questions of veterinary medicine: who deserves care? Who will get it? What do veterinarians know about the myriad species that fall under their purview? What technology is available to assist the doctor in diagnosis or treatment?

### Engaging With and Enlarging the Medical Humanities

A key focus of this article is on how the rise of veterinary anthropology, or the anthropological study of veterinary medicine, can connect with the already well established fields of (human) medical anthropology and/or of the broader medical humanities. The latter is that branch of knowledge dedicated to understanding the social meanings, practices, and institutions constituting and addressing culturally specific notions of human health and illness^2^. Although developed differently in various national contexts, the medical humanities often includes (or is in conversation with) medical anthropology, with its methodological emphasis on ethnographic research^3^. It also draws on related fields and methods from bioethics, philosophy, narrative analysis, and visual representation to build complex understandings of human health in its discursive, conceptual and experiential dimensions^4^.

Developing roughly since the 1970s, this interdisciplinary field now supports a robust body of scholarship, including that in medical anthropology. In the last decade or so the field has been expanding to the broader category of “health humanities” to de-center the role of physician-focused medical knowledge and to encompass the broader range of health practitioners from nurses to therapists to care-givers, and to draw more fulsomely on anthropology, linguistics, and literary theory. In the United States at least, veterinary medicine has largely remained outside the purview of medical anthropology, and medical/health humanities scholarship [important exceptions include works on the history of veterinary medicine and some work on OneHealth—the idea that human, animal and ecological health are intertwined, as in the case of zoonoses. See, for example: (15–22)]. Yet some of the key questions animating the fields of medical/health humanities are directly relevant to veterinary anthropology.

These questions include the following: What constitutes “health” and “illness?” Who gets to say so? How do these notions vary over time and among communities? How do such culturally specific definitions constitute subjects/subjectivity and social formations of difference? How is access to care apportioned? With what effects? What is the role of representation, including narrative (how we speak about illness) and other forms of discourse, like the visual arts and literature, in shaping attitudes toward care? How are cultural values played out in the medical realm? In addition to the critical and analytical tools these approaches supply, the health humanities also ask: What role should anthropology and the humanities play in medical training?

What affordances for our understandings of veterinary medicine might the anthropocentric medical humanities offer us for the development of veterinary anthropology? What limitations might inhere in its applicability? In return, how might including veterinary medicine/veterinary anthropology in the medical humanities pose productive provocations to our assumptions about medical worlds, and about what counts as medicine and who counts as a patient, calling for rethinking of key issues like doctor-patient relationships, agency, consent and refusal, embodiment and technology [see (23–25)]? To approach these questions, I will start in the veterinary clinic.

## Veterinary Medicine and Human Medicine: Charting Similarities and Differences

The practice of veterinary medicine in general, and the space of the clinic in particular, enacts a concentrated *relationalit*y across species lines, and the stakes in those relations can be as high as life and death. Veterinary medicine is produced in and through a matrix of ethics, knowledge, responsibility, and I want to suggest, to some degree of *incomprehensibility* between human and non-human animals, all anchored in a particular historical moment and within specific human communities. These cross-species relations are further enmeshed in the institutionalized structures of medical practice, and these vary widely from community to community, nation to nation. I restrict my remarks here to clinical practice in the contemporary United States, while acknowledging that animal doctoring is done around the world in different ways and by different types of specialists or lay practitioners.

Veterinary clinical medicine is both like and strikingly unlike human medicine in crucial ways. Certainly, there are deep similarities. Lab animals serve as research subjects to develop medicine for human benefit. Certain techniques are developed on animals prior to translation into human treatments. At the cellular level, for some species at least, basic biological processes are the same. And sometimes animals benefit from treatments that first become widespread for humans, as in the newer applications of radiological treatments for cancers in dogs. But in practice, what can appear so similar on the surface, from the white coated doctor to the MRI machine, is actually different from human medicine in almost every dimension from diagnosis to treatment to finances to ethics to technology. Understanding those differences and similarities is part of opening the medical humanities (including anthropology and sociology) to the more than human world.

Let me start with a case study, that of Cubby, the snake with heart disease, whom I met in 2018. Here the outer appearances are the same as with a human exam: white coats, spotless exam room, technology at the ready. Very quickly differences become apparent.

### Cubby the Snake With Heart Disease

One of the problems in treating a snake for heart disease is that you have to be able to find his or her heart. You'd think this would be easy, as the heart is an essential organ for any being who has one. But in the case of a snake, the heart lies not just below the head and neck, as is the case for most mammals, but rather about a third of the way down the body. Finding its exact placement even with a Doppler listening device can be tricky. I've seen experienced zoo veterinarians struggle to hear the heartbeat and to count its slow pace. Of course, the snake is not helping. Rather than lying still so the doctor can carefully place a stethoscope, the lithe being is twisting and turning in on herself with surprising strength, succeeding in moving the elusive heart ever away from the seeking instrument like a shell game—now you see it, and now you don't.

In this bi-species tango and tangle, the doctor and patient try to outwit each other, eyes locked each on the other in what appears to be a battle of wills. In the 2018 encounter I record here, the snake—a beautiful 4 foot rainbow boa we'll call “Cubby”—ultimately wins. The doctors do not succeed in hearing her heart, and instead have to settle for a visual approximation of heart beats, and they fail to draw a blood sample from the vena cava, the large vessel by the heart. The snake's symptoms included: incomplete shedding, rose colored spots of unknown origin on the glistening pearlescent abdomen, and lethargy. The snake was just not “acting like herself.” With little to go on, the snake is sent home with wide-spectrum anti-biotics, and we hoped for the best.

In this one encounter we see some of the ways that the veterinary clinic provides a privileged site of investigation of human relations with animals and for multi-species ethnography. For us as ethnographers, such a “fieldsite” brings all the daunting challenges of doing multi-species ethnography (26) to the fore as we try to understand its networks of human and non-human actors and technologies, all perceived by differing species-specific sensoriums^5^.

As Foucault has shown us, the clinic is a site of social, political, and epistemological conjunctions—a site for the production of knowledge and of subjects (33). But what types of knowledge, and what types of subjects? In the case of veterinary medicine, the clinic unites “patient” and “physician” across species barriers and challenges us to grapple with a specific mode of providing medical care where, it is assumed, the patient cannot “speak” and his/her subject position (as “animal”) is always already subordinate to that of the (always “human”) medical professional. In reality of course the non-human animal is an agential being and the veterinarian, just like an animal's owner/guardian, is often highly attuned to the ways in which the animal communicates affective states like fear and anxiety or physical states, like pain. A fundamental question for anthropologies of veterinary medicine is one that is, on the surface, so simple: What is the status of the animal in veterinary medicine?

## Mapping the Status of the Animal in Veterinary Medicine

To understand the formative impact of this question, I have conducted participant observation fieldwork over several years in several veterinary settings, starting in 2012^6^. I've sat in on lectures on body systems structure and function, participated in anatomy labs, and stood rubber booted on the necropsy floor as the huge intestines of a horse roiled out onto the concrete. I've learned to do a physical exam on a fish, and to identify clinical signs of animal abuse used by forensic scientists, and been coached in how to “break the bad news” of impending death to fearful pet owners. I've vaccinated chickens, taken the temperatures of goats at a zoo, helped collect semen from a bull on a research farm, and even assisted in wildlife surgery. In each of these settings, a different matrix of ethics, obligations, and practices enmeshed human and non-human animals, “doctors” and “patients.” It became important to ask: what difference does it make that the patient is a dog…. or a cat, or a horse, or a chicken, or an elephant, or…. a snake?

Two aspects are salient here: what difference does it make that the patient is a non-human animal rather than a human one? And, what difference does it make that the non-human patient is of a specific category of animal? (And here “category” can refer either to species or to a specific relationship to humans in a particular historical/cultural setting.)

In calculating this medical valence of difference, we always implicitly do so in relation to human medicine, the dominant, unmarked medical category. The mere presence of the modifier “veterinary” added to the word medicine, indicates the secondary nature of the field. Even the U.S. professional degrees are marked in this way…. A DVM, is a Doctor of Veterinary Medicine. An MD is simply a “medical doctor, ” not a Doctor of Human Medicine. The categorical distinction is also a hierarchical one, echoing the secondary status of animals *vis a vis* humans, and it has impacts on every dimension of clinical practice.

Using this binary frame of human medicine/animal medicine assumes that the dividing line between “human subjects” and “animal subjects” is a clear and absolute one, and we know that this is not true. Instead the line of demarcation between the human and non-human realms is historically and culturally shaped, contextually invoked, and differentially impactful in distinct realms of practice, including scientific, religious, or spiritual or imaginative and artistic realms. But in the contemporary U.S. veterinary medical world these distinctions are presumed to be quite straightforward^7^. Children go to pediatricians, while puppies go to veterinarians. No one brings worms to the veterinarian for a checkup, except inadvertently.

Within these widely accepted boundaries of “who” and “what” counts as an animal and hence as a candidate for veterinary medicine in the U.S., key dividing lines show how human socio-cultural understandings of particular human-animal relations are mapped complexly onto species lines. One key dividing line is determined by human ownership. With some exceptions, wild or feral animals rarely see a veterinarian— most veterinary efforts are directed toward animals who are owned by humans^8^.

### Categories of Practice and Differential Valuations

In its professional training systems, contemporary U.S. veterinary medicine is divided into the following categories of training and practice: production medicine (which focuses on those animals raised for food); companion animal medicine, which focuses on companion animals like dogs and cats; equine medicine; and exotic/wildlife/zoo medicine, which focuses on more unusual pets, like Cubby the snake, or parrots, fish, and rabbits, and caring for injured wild animals, or captive animals in zoos.

Some species cross these dividing lines of course…. a pet chicken will come in through the exotics clinic, while a chicken who is one of a flock of ten thousand on a poultry farm will be treated as part of a flock by a production veterinarian, with the goal of protecting herd health. Differential valuations according to human categories of use and affection forcefully shape the medical starting points and outcomes for individual animals. They determine what type of specialist will see the animal, inducting the animal into a specific matrix of normative assumptions about care. And it all starts with an examination of the animal.

For those animals who do come into the orbit of clinical veterinary medicine, the examination can take place out in the open, on a farm if a sick cow is down in a field, or in a wildlife preserve if, for example, a vet is performing a vasectomy on an elephant, or in a barn to examine a racehorse. Within this wide range of sites of practice, I want to focus on the encounters that take place in a U.S. veterinary hospital, where the meeting takes place in a human-controlled space, complete with white coats, gleaming, sterilized instruments, high-tech diagnostic equipment, and fee schedules for each and every procedure from nail clipping to cancer chemotherapy. These are the sites where the outward parallels with human medicine are strongest.

At every step of the way, vets must get approval from clients to do anything: to do diagnostic tests, administer treatments, order drugs. Legal frameworks of consent are invoked, to protect the doctor. Consent is performed as a human to human contract, but as Megan Donald has argued in her article on ethics and geographies of affect, “By being there then, the non-human animal is always a part of the communication and decisions around consent have the potential to be co-constitutive” [(34), p. 5]. We saw this explicitly in my opening example of Cubby the snake who did not consent to having her heartbeat monitored in the only way humans could hear it.

In the U.S. the types of treatment the animal receives is directly related to the amount of money spent at each step of the process, and few have pet health insurance. For veterinarians this means that they are often restricted from practicing what they know to be their best medicine. For the animals this often means less precise diagnosis. Some tests are expensive, and without them the animals may have to receive less targeted treatments. For the owners, it often means struggling with a calculus of competing values and competing financial needs in what I am calling “a moral mathematics of care.” Money is a constituent component of the practice of veterinary medicine, and its financial infrastructure is one of the key differences between human and animal-focused medicine.

Despite the fact that there is no universal health care in the U.S. resulting in vast disparities of access to health care, even those humans with no money at all may receive limited treatment either through government subsidies, such as Medicaid, a program for the indigent, or through philanthropic funds at hospitals. No one with a life threatening medical situation arriving at the emergency room of a human hospital can be turned away simply because they lack insurance^9^. They will at least receive basic assistance even if not granted access to the most elaborate regimes of care.

But in the case of U.S. medicine, the situation for humans and animals is different. The obligations of both doctors and animal owners do not demand that treatment be sought or given. Veterinarians can legally turn away animals brought into their clinic—for example, a dog found by the side of the highway—if the person bringing that animal in cannot pay [a case study that often appears in veterinary textbooks, see (10, 35)]. And, as an “owner” even if my pet has a serious illness, I can legally reject treatment if I do not have the money to pay, or if I just choose not to spend it. I can instead opt for a lesser treatment, perhaps some pain medicine, and still be in compliance with anti-cruelty statutes. Most veterinary care is optional.

And what of the “un-owned?” Unlike human medicine, this is a crucial divide between categories. An orphaned child presumably becomes a ward of the state which has an obligation to provide minimal care at least, including medical care. This does not always happen, but it is at least the statutory presumption. For “wild” or “feral” animals, there is no such presumption. Many of the medical concerns directed toward unowned animals are corralled into the zone of zoonoses threats and epidemiology.

Outside these conceptual categories of the pet and the wild lie the owned animals raised for food or their products: beef and dairy cows, sheep, pigs, chickens, and so on. Here medical care is most often conceived of as “herd health” and the status of the individual as “sick” or “well” is more likely to be understood as “productive” or “non-productive.” A cow with an eye abscess can still produce adequate and safe milk for the dairy industry and, as this is not a communicable disease that could spread through the herd, some farmers may choose to forego the cost of veterinary medicine to alleviate her eye illness.

The status of animals within veterinary medicine is thus both determined by larger culturally specific social valuations (of valuing pigs over grasshoppers, for example) and by an internal matrix that recognizes the role of owners and ownership in the practice of medicine, from those owners who have the money to spend on care for their pet and choose not to, to those who would rather go hungry themselves than see their pet suffer from lack of medical care, as sociologist Leslie Irvine has documented in her U.S. studies of homeless individuals and their pets (36). From the chick that is one among 10,000 in a poultry barn to the pet rat brought in for mammary tumor surgery, the status of each animal is a complicated calculus directly related to the care he/she will receive.

## The Physiological, Technological and Epistemological Triumvirate

If the practice of medicine for animals is complexly shaped by issues of ownership and money, it is equally constrained by the interplay between the cultural valuation of species and the available knowledge about the medical needs of that species. Remember that in human medicine research is conducted to help one species: the human. We are now aware of some of the crucial limitations of human medical research, in terms of under-representing women in research projects and of inadequately accounting for health differentials among various ethnic and racially marked populations. But still the overall species is the same. In veterinary medicine, the number of species to be treated is astounding—from pigs to parrots, from capybaras to cobras. Indeed, this is a point of pride among veterinary students who, chafing against their sense of a second-class status in public culture compared to doctors for humans, wear tee-shirts emblazoned with the slogan “Real Doctors Treat More than one Species!” (37). Simultaneously, however, this virtually limitless variety of animal patients is also a source of challenge in terms of available knowledge.

### Knowledge Bases: The Known, Unknown, and the Unknowable

Within the above listed categories of medical practice, the exotics veterinarian—who deals with those animals who fall outside of the production animal strain, and beyond common companion animals like dogs and cats, and horses– struggles most dramatically with questions of evidence: its definition, gathering it, and interpreting it. Huge swaths of the unknown unfurl. Baseline studies have not even been done on the normative blood values for many species. Diagnostic procedures may be impossible to implement (remember how hard it was to get blood from that snake?), or technically poorly suited for animals.

The physiological, technological, and epistemological triumvirate comes together in veterinary medicine in particularly challenging and frustrating ways in part because so often the protocols have to be adapted from human medicine in a re-tailoring that rarely fits like a glove.

Clever veterinarians work their way around these limitations, using drugs in unconventional ways, or retrofitting human technologies to fit non-human patients. For example, at my University, radiologist Dr. Robert O'Brien designed the “VetMouseTrap” for use in imaging. A clear plastic tube snuggly holds the feline in a static position as he is positioned on a CT scan machine designed for humans, helping technicians obtain a clear image. Unlike a human, of course, the cat cannot simply be told to “lie still.”

The one substantial exception to this second-classness is the ability of veterinarians to legally offer euthanasia, “a good death, ” to suffering animals, an option largely denied human physicians (14). Euthanasia is a complex terrain and often a high stress one for veterinarians, who face requests for “convenience euthanasia” from owners, as well as owners who refuse this option even in the face of extreme suffering. But it is one of the distinctive differences with human medicine, and one which is allowed for animals due to their own second class status, that is, the very fact that they are not human.

Within this larger cultural calculus of value across multiple communities and in relation to multiple species, veterinarians also face another gulf of unknowability between our species and others—the lack of narration. Remember that the patient cannot give us his or her medical history verbally. He cannot narrate the incident that befell him. Often, neither can his human companion. Did the dog eat rat poison in the neighbor's yard when he was off exploring? Who knows? Has the octopus been feeling nauseated since Sunday? How could we tell? Even mammals, with bodily systems more parallel to our own, may stoically hide signs of illness that, outside of captivity, might make them easy prey.

In this question of self-narration or narration by a caretaker, we do have a parallel in human medicine with very young children who can't speak or others who for reasons of mental or physical restriction cannot narrate their own sense of how they feel or what took place [for example (38–40)]. But in those cases at the very least, the physician can usually read signs of pain in a bodily system like his or her own, or easily conduct some baseline tests for organ function. For many animal patients, such tests are out of their owner's economic reach. And only recently have codified renditions of markers of pain for some species, called “grimace scales, ” been introduced into veterinary medical training to help clinicians estimate the degree of pain the animal is experiencing.

And, finally, for so many species and so many individual animals, the clinic must be a space of fear. As I watched a veterinarian try to examine an eagle's reconstructed wing for healing, the vet had to grab the giant bird in a blanket, clutch him to her chest facing outward to avoid the sharp talons and potentially eye-piercing beak, and somehow test range of motion on the healing feathered limb. The bird's eyes were wide, his body heaving, and his beak opening and closing in clacking sounds. This is an agential subject caught literally in the web of medicine and fighting to get away.

Thus, medicine across the species lines, practiced in so many parts of the U.S. today in the space of a clinic that looks very much like a human medical clinic with its white coated doctor and sterilized instruments, is actually more different than we might at first imagine. Returning to my earlier question: what difference does it make when the patient is a dog (or a…..)? It makes a constitutive difference in every aspect of veterinary medicine—from access to care, to diagnosis to treatment.

Given this fundamental fact of veterinary medicine, one of the challenges of conducting ethnographic research in a veterinary clinic is similar to the challenges faced by veterinarians everyday: to imaginatively construct the encounter from the animal's point of view and from the animal's particular sensorium and experiences—which gets harder and harder to imagine as the bodies become less and less like our own.

#### Veterinary Anthropology: The Challenges of Redesigning Ethnographic Research

In their recent 2017 book, Ethnography after Humanism: Power, Politics and Method in Multi-species Research, authors Hamilton and Taylor (41) embrace the challenges of expanding the practice of ethnography to include communities comprised of relationships between more than one species, urging us to pay attention to sensory and motion-based dimensions of life. Those dimensions are crucial, I believe, in rendering the materiality of non-human animal lives.

But the multi-species frame is not just important for human-animal studies, it is also of signal importance for the contemporary practice of ethnography itself, that critical research and writing strategy with origins in anthropology that is conventionally based on direct engagement—especially fieldwork—with communities over substantial periods of time—a research method which since the mid 1980s has been increasingly self-reflexive about issues of power dynamics, its linkage with colonial histories, and its own representational strategies, and embracing efforts at “decolonization” of the discipline^10^. Ethnography is increasingly used in other fields too, like sociology, geography, and communication studies, among others. What could this new commitment to conducting ethnography across species lines look like for studies of veterinary medicine?

In trying to focus on the experiences of non-humans we face many and at times insurmountable barriers, but the first step is to craft a multi-focal approach. While watching what the doctor is doing and saying, and not saying, I must also attend not only to the owner if he or she is present, but to the physical, vocal, visual and even aromatic responses of the non-human in the examination room or treatment suite. Let me share an example.

### “Herbie” the Bearded Dragon

Let's look at a medical encounter from the perspective (as best I might imagine it) of a lizard…a juvenile bearded dragon, say. These desert dwelling reptiles with striking markings have recently become more popular as pets in the US.

On June 2, 2017, a juvenile bearded dragon of a light sandy color with darker chocolate markings, and just three inches long in body and another three in tail, was brought into the clinic (again) by a local pet store employee. Let's call him—the reptile—“Herbie” which is close to his name but not exactly. Herbie arrived in a small clear plastic container pocked with breathing holes. As a being owned by a store, Herbie is a commodity, one whose status as a “pet to be” underwrites the financial investment in his care.

“Herbie” had been seen once before a couple of weeks ago—he was not growing at an appropriate rate for his age. X-rays, what vets call “radiographs, ” revealed an alarming lack of bone. A diet poor in calcium had resulted in front limbs that were so weak that they often lay splayed behind him, like swimming fins, as he scooted around on his stomach. His lower jaw bent during an exam, in a way that bone should never waver. Somehow he remained active. The vets prescribed calcium.

So now Herbie was back for a recheck X-ray 2 weeks later, and this is where the comedy and pathos came together in this ethnographic multi-species “contact zone” moment (42–44).

Imagine an X-ray machine suspended over a table the size you would lie on, and then imagine an animal the size of one of your fingers, who has no intention of standing still. Herbie is surrounded by humans, giant in relative size. They are grasping him in one hand, and then, to keep him from escaping during the procedure, taping him down on a big foam rubber block to position him under the beam of the X-ray machine. Even this doesn't really work technically. He's just too small, so the radiology technicians suggest putting him in another room—the one they use for horses (!)—so they can use a more mobile X-ray machine. There, squatting on the cement floor, wrapped in a lead apron, one of the fabulously competent veterinary technicians tries to position Herbie on another slab of foam.

Just then, someone notices the large floor drain a mere foot away—a gaping maw of steel bars a foot square—“Don't let him escape down the drain!” she shouts. At this, howls of laughter escape from all the students watching as the comedy and pathos of it all engulfs us…giant humans struggling to contain a tiny patient in a room sized for horses, deploying technology fitted for a different species, while working against every escape impulse that Herbie must have had triggered by the situation, surely incomprehensible to him in terms of his daily life in the pet store up to that point.

After all the taping, the laughter, and the rotations, Herbie is done and returned to his bare plastic carrying cage. He is safe, did not escape down the drain, and, although surely disoriented from the rotations and perhaps frightened at the event and restraint, physically he is none the worse for the wear. The vets seem relieved, having managed to negotiate their diagnostic needs with Herbie's actions and apparent desires.

In the end the news for Herbie was encouraging. Herbie has a chance. But how can we really understand his experience there? For some species, we have cortisol measures of stress, or a relatively reliable understanding of their body language (ears back, snarling) that indicate fear aggression, but with Herbie, like many other animals, we know little and some may even presume there is little to know—little of an inner life or consciousness, little subjectivity, few outer signs of response.

In attempting a multi-species ethnography we have to confront our ignorance, but not let it paralyze us. I may never understand Herbie's world, indeed can only attempt to do so through the by-definition anthropo-centric frame of my own human experience of the world. But I can turn my ethnographic gaze equally on him as well as the human actors in the space, giving him my deep attention, as scholars such as Haraway have invited us to do (3, 45). In that attention—that act of attending to—I attempt to position him in my research as a subject in a multi-subject set of actions, an actor in a complex network of actors, events, times, and places, and a rhizomic map of shifting connections and logics (46–48).

Ultimately as humans we can only understand the lived experiences of non-humans through *radical acts of empathy and imagination*. This, I argue, can and should be one of the constituent components of veterinary anthropology. Such acts will always, by definition, fail. Our abilities to grasp the ways that other species experience the world are necessarily limited by our own anthropocentric ways of being and knowing, by our own sensoriums and cognitive capacities [see (49, 50)]. In this sense, such a prescription is less about specific acts than about attitude; it is, we might say, a politics, a politics of recognition.

But this is precisely where the cultivation of imaginative empathy can serve us, both in our wider relations with non-human animals, and specifically in the context of practicing veterinary medicine. Cultivating these capacities is a new frontier for veterinary education (51) and a new dimension for the medical humanities which already emphasize the ability of the arts and humanities to cultivate visual acumen, interpersonal empathy, and attentive listening to the narratives of human patients [see (9)]. Several new areas of research can emerge at this intersection of veterinary anthropology and the medical humanities. While some will only reveal themselves once our veterinary anthropology initiatives are more fully underway, others are already coming into focus, especially in the realms of agency, consent, and narrative.

### Subjectivity and Agency, Consent, and Doctor-Patient Obligation

In centering the animal as agentive subject, we embrace the challenge of articulating the limits of our understanding of what the veterinary encounter might be like from the animal's point-of-view. The animal moves from being the object of medical intervention to the subject of medical care.

In the US veterinary world, two distinct arenas currently recognize this sense of subjectivity without necessarily using the notion of an “agential subject” to conceptualize the animal patient. These are the development of “low stress” handling techniques, promulgated since the mid 2000s by Dr. Sophia Lin and others (https://lowstresshandling.com), and on the other hand, attempts in lab animal medicine to recognize visual signs of pain and distress through the development, in the last decade, of what are called “grimace” scales, mentioned briefly earlier (52, 53).

The former, low stress handling techniques, include a soft embrace to stabilize the animal, quiet exam rooms, timing the pacing of the exam to the tolerance of the animal, with frequent breaks between procedures, covering the eyes, wrapping the body to limit struggle, and attending to discomfort in the exam, for example, when doing a rectal exam on a dog, avoiding cranking the tail up high just for clinicians' ease, which can wrench the tail. And of course, frequent treats are a must!

One may wonder why all clinical techniques are not already “low stress” when possible, but all of this can cost money. In applying these techniques the small animal clinician may take longer on each patient's exam, and many practitioners are tightly scheduled with a new patient every 15 mins. However, in the long run such techniques “pay off.” They can result in return clients for whom vet visits are no longer an exercise in futility and struggle, and by relieving stress between clinicians and owners, can result in greater compliance with health care protocols by pet owners, and so ultimately yield better care for the animal. By centering the animal as agential subject, we simultaneously improve the animals' health care.

Grimace scales function differently, in that they are used, primarily in lab settings, to assess the amount of discomfort or pain an individual of a specific species is feeling. This might impact the amount of analgesic offered, or the duration of an experiment. Here a knowledge of how to read non-human faces, like those of mice or rabbits, is key. Prey animals often hide signs of pain that could make them vulnerable, so the scale helps lab workers read more subtle signs of distress like tightening around the orbit of the eye, nose and cheek bulge, ear position, and whisker position change. It provides, in a sense, a translation of physical evidence of subjective response from one species (the mouse) to another (us). New research by Katharina Hohlbaum et al. (54) shows that experienced observers are better at reading these signs accurately, so enhanced training in assessing pain should be integrated into veterinary education. Doing so recognizes the animal as a communicative being and challenges veterinary professionals to enhance their ability to accurately interpret those communicative signs.

This idea of visual interpretation of pain was adopted from human medicine where scales for evaluating facial expressions and body language have long been used for non-verbal humans, especially in pediatrics, which uses the NFCS—Neonatal Facial Coding System, developed in the late 1980s, for example. It was only in 2010 that such scales were developed for animals, but now grimace scales are available for ferrets, rabbits, cats, sheep, pigs, and horses.

Complementing this research focus on the animal as communicative, agentive subject, a second area of ethnographic research could lie in analyzing the notion of “consent.” Consent is usually performed as a human to human contract, but as Megan Donald (34) has argued, the co-presence of the non-human animal at decision making time implies that consent decisions have the potential to be multi-species events (We saw this explicitly in my opening example of Cubby the snake who was not consenting to start her exam). Similarly, in a different context of UK lab animal research, Gail Davis et al. have posed the question of whether animals can consent to be research subjects (55, 56).

In addition, new research on consent in the UK by veterinarian and scholar Carol Gray demonstrates that consent is under-researched in veterinary medicine and that clients generally perceive it to be merely legal protection for the doctor (56, 57). She argues that consent forms should create the conditions for and document fuller discussions of procedures and risks, and provide protection for client, patient, and doctor. She calls for renewed attention to the question of balancing client autonomy and patients' best interests. Here the animal, while willingly participating or not, and thus indicating some level of consent, has his or her interests represented by the owner, who has legal standing while the animal does not. The parallel here in human medicine is children and those who are mentally incapacitated.

Ethical concerns arise here in part due to the financial status of veterinary care, care which in most cases is voluntary. Within the limits of animal cruelty statues, care can be refused. Thus in the issue of consent, veterinarian and ethicist Simon Coughlin in Australia has argued that small animal veterinarians should become not simply medical advisors, but “strong patient advocates”—a role that is less necessary for human patients when human health is seen as a public good and often—at least in theory— paid for by the state or by private insurance, even if those benefits are as we know unequally available. Veterinarians, he argues, often face insidious pressures NOT to treat patients, and thus should expand their sense of professional obligation not only to *treat* but *to advocate for* the treatment which is in the best interests of their patients (58) [see also (59)]. This in effect recalibrates the triangular relationship between animal/owner/and doctor, with the primary ethical relationship now being between animal and doctor.

In a quick final example, we see this changing notion of relationship reflected in the various oaths veterinarians take in different countries. As Bones and Yeates (60) demonstrate, the veterinary profession conceives of its obligations to animals, to humans, and to the state differently in different countries. They note, “Oaths are social structures, and each country's oath is situated within the historical context of the veterinary profession in that society.” “…[and] Oaths are solemn affirmations of a belief or a pledge to adhere to a certain standards of behavior.” [(60), p. 20]. I would add that oaths encode a complex understanding of appropriate and desirable relations between human and non-human animals. Veterinary practice is thus always a performative ethics.

In the U.K. veterinarians must be members of the Royal Society of Veterinary Surgeons, and their oath reads: “…. my constant endeavor will be to ensure the *welfare* of animals committed to my care.” [(60), p. 29].

In Brazil, veterinarians confirm a complex oath promising to “balance the benefit of the health and welfare of animals, with the quality of their products and the prevention of zoonoses, … the promotion of sustainable development, the protection of biodiversity and the …. balanced progress of human society.” [(60), p. 31].

In the U.S., the American Veterinary Medical Association oath obligates one to use their skills and knowledge to benefit society through the protection of animal health and welfare, and also calls on practitioners to prevent animal suffering, conserve animal resources, and promote public health and the advancement of medical knowledge [(60), p. 26]. The latter implicitly refers to the use of animals in human-centered research—indicating that it is deemed necessary to balance animal welfare and human benefit. It was only in 2010 that the word “welfare” was added in the phrase “the protection of animal health *and welfare*” (my emphasis), and then only after vigorous national debate. It is remarkable that a commitment to animal welfare could be controversial, but some vets feared criticism of industrial farm animal medicine would result.

An expanded version of veterinary oaths could emphasize a commitment to the development of skills in cross-species communication and empathy.

## Conclusions

Throughout this article, I have drawn on anthropological fieldwork in clinical settings to refine an understanding of how contemporary veterinary medicine is organized and enacted in the U.S. in both its institutional structures and in daily practice. I have suggested that the veterinary clinic forms a privileged site for anthropological research, because it concentrates and enacts, in clear and high stakes life-and-death ways, specific sets of relations between human and non-human animals which are revealed in performative practices. These practices in turn reveal the social construction of value and of relations of obligations, understanding, incomprehensibility, and possibility across species lines.

I have argued that long standing work in the medical humanities and in medical anthropology can provide a trenchant seam of interlocutory provocations for us as veterinary anthropologists as we approach our work. In return, the expansion of the medical humanities to include veterinary medicine can fruitfully challenge extant notions of doctor-patient relations, of bioethics, and of what constitutes health (61, 62). Furthermore, I have suggested that the questions that engaging with such work can raise for us—questions about agency, consent, ethical obligations, anthropocentrism, the production of knowledge and of cultural value—have implications both for the practice of veterinary medicine and its training curricula.

Ultimately, if we are to better grasp the ways in which veterinary clinical medicine—not simply in theory but in its complexities of performative practice—constitutes a particularly concentrated set of relations between some humans and some non-human animals, we must be able to situate Herbie and Cubby and all the others as active agents in our narratives and our analyses. This demand asks us to reconsider issues like consent and the relationship between patient and doctor, and forces us to stretch our concepts of narrative and medicine. It invites us to cultivate acts of radical imagination and empathy, and to expend more energy attending to non-human's bodily behavior and sensory modes of apprehending and acting on their worlds. Doing so can help reveal how cultural value, scientific knowledge, and the performance of compassion and care come together in complex and at times conflicting ways in the space of the veterinary clinic, articulating both the “human” and the “animal.”

Working at the productive seams of anthropology and the medical humanities, both anthropologists and veterinarians can ask what happens when some of the key foci of that medical humanities work is addressed in veterinary clinical settings. How do we conceive of the category of “patient” when that being is non-human? What structures of valuation—from the cultural to the financial—frame the degree of individuation accorded each animal in the medical context? Who is deemed worthy of what types of care? Just how central is the position of “the animal” in veterinary medicine? To what degree is this field of medicine for animals profoundly anthropocentric? How can we understand, and narrate, animal subjectivity in ways that can counter that anthropocentricity? What enhancements to veterinary training might the medical humanities bring in terms of cultivating cross-species empathy through radical acts of imagination?

And finally as anthropologists working in multi-species veterinary worlds, how might the questions we ask and the types of evidence we grapple with gathering ultimately enhance the lives of both human and non-human animals?

## Author Contributions

The author confirms being the sole contributor of this work and has approved it for publication.

## Funding

Portions of the field work that provided the basis for this article were monetarily supported by the University of Illinois Research Board, specifically for fieldwork at Colorado State University College of Veterinary Medicine in fall of 2018 and for discussions at the University of Edinburgh in spring of 2019 (Award #18147).

## Conflict of Interest

The author declares that the research was conducted in the absence of any commercial or financial relationships that could be construed as a potential conflict of interest.

## Publisher's Note

All claims expressed in this article are solely those of the authors and do not necessarily represent those of their affiliated organizations, or those of the publisher, the editors and the reviewers. Any product that may be evaluated in this article, or claim that may be made by its manufacturer, is not guaranteed or endorsed by the publisher.

## Footnotes

^1^I explore these relationships in my book-in-progress, Medicine Across the Species Line.

^2^For sample overviews, see Jones et al. (5), Cole et al. (6), and Crawford et al. (7). Several leading medical schools like Duke University and Yale University have medical or health humanities programs as part of their curricula, and others have associated centers or initiatives, such as the Institute for Bioethics and health Humanities at the University of Texas Medical Branch in Galveston, Texas.

^3^Medical Anthropology is a vigorous subfield within the larger field of anthropology. The Society for Medical Anthropology is a formally recognized Section within the American Anthropological Society, for example, and includes members from around the world, including scholars in Europe and elsewhere. A sample text providing an overview is: Brown and Closer (8). A leading journal is the *Medical Anthropological Quarterly*.

^4^See Charon et al. (9) for an overview of narrative medicine. See also *Synapsis: A Health Humanities Journal*, an online journal founded in 2017 and associated with the Institute for Comparative Literature and Society at Columbia University, N.Y.C. (www.medicalhealthhumanities.com). Veterinary medicine is underrepresented in bioethics work in comparison with human medicine, but the work of philosopher Bernard Rollins has been robust and key in shaping some of the terms of debate in veterinary medicine [for just one of his many books; see Rollins (10)]. A small but dedicated organization, the Society for Veterinary Medical Ethics (www.svme.org), founded in 1994, provides a hub for intensive ethical debates. See also, Linzey and Linzey (11). In addition, work in “feminist ethics of care” has intersected with human-animal studies in important ways. For just two key texts, see Donovan and Adams (12) and Gruen (13). Euthanasia is another key issue for ethical decision-making by veterinarians. See Morris (14).

^5^With a few key exceptions, on the whole veterinary medicine has received comparatively little attention from scholars in the social sciences and humanities. A key exception here is in social history and its intersection with the history of science. Works like U.S. historian and veterinarian Jones's (16) account of the rise of the veterinary profession and British historian and veterinarian Abigail Woods' (20) are among the important exceptions, as is work by veterinarian and social historian Andrew Gardiner and historian Neil Pemberton in the U.K. While it is beyond the scope of this article to supply a comprehensive bibliography of work on veterinary history, this is a rich field which, while as yet attracting only a handful of gifted scholars, has begun to make serious inroads into medical history. Previously, at least in the U.S., veterinary histories were often crafted by interested veterinarians who sought to retain the history of their discipline, especially through memoirs, but who largely lacked training in scholarship or historical studies (for example see the publications by the American Veterinary Medical History Society, founded in 1978. See www.avmhs.org). It is only more recently that the field has attracted the attention of professional scholars in social history, the history of medicine, or science and technology studies. For a few additional key studies, please see the following: Pemberton and Worboys (19), Gardiner [(15), p. 355–376], and Woods [(20), p. 115–131]. Similarly, veterinary medicine has largely been ignored in interdisciplinary animal studies, as well as in the medical or health humanities. Studies of One Health initiatives do provide an exception. For example see: Woods et al. (22), King (17), and Kirk and Worboys (18). See also the new sociologically based study of poverty and access to veterinary care (27). Legal scholar Braverman's article (28), spring 2018 is also relevant as it draws on interviews by the author. A brand new sociological study in 2022 by Vermiliya adds another social science contribution to the study of gender and veterinary medicine [see (29)]. In terms of curricular studies, works by folklorist Carolyn Ware and Nadine Dolby stand out as rare examples of work by scholars located outside of veterinary colleges. As a discipline, anthropology is increasingly embracing animal studies, with recent fieldwork based books focusing on both humans and animals in specific contexts, like Govindrajan's (30) and Salazar Parrenas' (31) and the rise of “multi-species ethnography, ” but the vast majority of those works do not concentrate on veterinary medicine. For a recent overview about the social sciences, see Bonnaud and Fortune (32).

^6^The observations I'm sharing in this chapter are based on several years of fieldwork (2012–2018) at my home institution, the University of Illinois College of Veterinary Medicine in Urbana-Champaign, for which I gratefully acknowledge the permission granted by faculty, students and administrators who have welcomed me into their world. In addition, I am grateful to students, faculty and administrators at the Colorado State University College of Veterinary Medicine for facilitating my fieldwork there in fall of 2018, and to colleagues at the University of Edinburgh Royal “Dick” College of Veterinary Medicine for crucial conversations there in spring 2019.

^7^I should mention here that the national structures of training for veterinarians differ substantially. In the US, veterinary training is not a 5 year BSVM degree as it is in, for example, the U.K. Rather it is a 4 year post-graduate degree following on a 4 year undergraduate degree. In addition there are substantial differences in the professional oaths of veterinarians from nation to nation, each oath encapsulating a variety of notions of what the relationship between doctor, animal and the national good should be.

^8^Veterinarians participate in TNR (Trap, Neuter, Return) programs for feral cats to control populations, and also in the emerging field of wildlife fertility control, which involves the use of both surgical techniques (ovariectomies, tubal ligations and vasectomies) and non-surgical methods (contraceptive vaccines) in the management of U.S. wildlife populations, like white tailed deer and free roaming horses (see for example the Humane Society Veterinary Medical Association webinar on “The Role of Veterinarians in the Emerging Field of Wildlife Fertility Control” offered September 15, 2021, by Drs. Randall E. Junge, Kathleen A. Carey, and Stephanie Boyles Griffin).

^9^I want to acknowledge here again the differences between nations: for example, in nations with a national health service, as in the U.K, this creates a different context of differentiation between human medicine and medicine for animals. The closest approximation in the U.S. to a public system of health support for animals would be free or low-cost spay and neuter clinics offered on a periodic basis in some communities, often by local Colleges of Veterinary medicine. Debates about inequity of access to veterinary care due to its privatization and cost are emerging in the U.S. See the special 140 page report “Access to Veterinary Care: Barriers, Current Practices, and Public Policy Report, ” by the “Access to Veterinary Care Coalition, ” December, 2018, from the University of Tennessee at Knoxville, College of Social Work. Many veterinarians donate their time and services to help clients in need. But more broadly the issue is moving into veterinary education *via* a discussion of “spectrum of care” vs. “gold-standard” treatments. The former acknowledges that the most targeted medical treatments are not always in reach for clients, and that veterinarians can still respond responsibly to those limits by considering a wider range of interventions which could benefit the animal even if a “cure” or complete return of function is less likely to result.

^10^For excellent resources on the project of “decolonization” in anthropology please see the UIUC anthropology department website www.anth.uiuc.edu. There you will find the “Guide toward Inclusive, Anti-Racist, Decolonial Teaching in Anthropology, ” prepared by our faculty Krystal Smalls, Jenny Davis, Cris Hughes, and graduate student Emma Versraete. In addition, it should be noted that “ethnography” as a research practice/methodology is a multi-valenced reference, and for some can consist mainly of interviews and surveys, while for others, those methods may be separate from or supplementary to “participant observation” which traditionally has implied an *in-situ* and lengthy embedded engagement with and collaboration with a community that the anthropologist is striving to understand in terms of its modes of social organization, cultural practices, and meaning making. Since the 1990s, the traditional notion of a single “fieldsite” has come under pressure and researchers have explored multi-sited research and even more recently, the rise of the internet has offered opportunities for “virtual” ethnographies as well.
